# CrossFit Overview: Systematic Review and Meta-analysis

**DOI:** 10.1186/s40798-018-0124-5

**Published:** 2018-02-26

**Authors:** João Gustavo Claudino, Tim J. Gabbett, Frank Bourgeois, Helton de Sá Souza, Rafael Chagas Miranda, Bruno Mezêncio, Rafael Soncin, Carlos Alberto Cardoso Filho, Martim Bottaro, Arnaldo Jose Hernandez, Alberto Carlos Amadio, Julio Cerca Serrão

**Affiliations:** 1 School of Physical Education and Sport, Laboratory of Biomechanics, University of São Paulo, São Paulo, Brazil; 2 Faculty of Physical Education, University of Itaúna, Itaúna, Brazil; 3 Gabbett Performance Solutions, Brisbane, Australia; 4 Institute for Resilient Regions, University of Southern Queensland, Ipswich, Australia; 5 Sport Performance Research Institute New Zealand, Auckland University of Technology, Auckland, New Zealand; 6 Department of Psychobiology, Federal University of São Paulo, São Paulo, Brazil; 7 College of Physical Education, University of Brasília, Brasília, Brazil; 8 Orthopedics and Traumatology Institute, University of São Paulo, São Paulo, Brazil

**Keywords:** High-intensity functional training, High-intensity interval training, Training load

## Abstract

**Background:**

CrossFit is recognized as one of the fastest growing high-intensity functional training modes in the world. However, scientific data regarding the practice of CrossFit is sparse. Therefore, the objective of this study is to analyze the findings of scientific literature related to CrossFit via systematic review and meta-analysis.

**Methods:**

Systematic searches of the PubMed, Web of Science, Scopus, Bireme/MedLine, and SciELO online databases were conducted for articles reporting the effects of CrossFit training. The systematic review followed the PRISMA guidelines. The Oxford Levels of Evidence was used for all included articles, and only studies that investigated the effects of CrossFit as a training program were included in the meta-analysis. For the meta-analysis, effect sizes (ESs) with 95% confidence interval (CI) were calculated and heterogeneity was assessed using a random-effects model.

**Results:**

Thirty-one articles were included in the systematic review and four were included in the meta-analysis. However, only two studies had a high level of evidence at low risk of bias. Scientific literature related to CrossFit has reported on body composition, psycho-physiological parameters, musculoskeletal injury risk, life and health aspects, and psycho-social behavior. In the meta-analysis, significant results were not found for any variables.

**Conclusions:**

The current scientific literature related to CrossFit has few studies with high level of evidence at low risk of bias. However, preliminary data has suggested that CrossFit practice is associated with higher levels of sense of community, satisfaction, and motivation.

**Electronic supplementary material:**

The online version of this article (10.1186/s40798-018-0124-5) contains supplementary material, which is available to authorized users.

## Key Points

- For a large majority of studies, a low level of evidence and a high risk of bias were found. There is a need to improve the methodological approaches in further studies.
- In the scientific literature, there is a gap to be filled in the area of controlling training load. Given the importance of managing training load in reducing injury risk and optimizing athletic performance, these approaches could be used to support CrossFit practice.
- Initial reports of higher levels of sense of community, satisfaction, and motivation during CrossFit training were found in the scientific literature.

## Background

CrossFit is recognized as one of the fastest growing modes of high-intensity functional training. According to the official CrossFit website (map.crossfit.com), CrossFit boxes are located in 142 countries across seven continents with more than 10,000 affiliates [1]. This strength and conditioning program is used to optimize physical competence in ten fitness domains: (1) cardiovascular/respiratory endurance, (2) stamina, (3) strength, (4) flexibility, (5) power, (6) speed, (7) coordination, (8) agility, (9) balance, and (10) accuracy [2]. CrossFit training is usually performed with high-intensity, functional movements called “workout of the day” (WOD) [3]. In these training sessions, high-intensity exercises are executed quickly, repetitively, and with little or no recovery time between sets [4]. With the focus on constantly varying functional movements, CrossFit training uses the main elements of gymnastics (e.g., handstand and ring exercises), weightlifting exercises (e.g., barbell squats and presses), and cardiovascular activities (e.g., running or rowing) as exercise tasks [5]. According to Glassman, who is the founder of CrossFit, the methodology that drives CrossFit training is entirely empirical. Furthermore, Glassman described that “meaningful statements about safety, efficacy, and efficiency, the three most important and interdependent facets of any fitness program, can be supported only by measurable, observable, repeatable facts, i.e., data” [3].

CrossFit is also considered an option for high-intensity interval training (HIIT). Consequently, HIIT has become one of the top 3 worldwide fitness trends since 2013 according to the American College Sports Medicine (ACSM) annual survey [6–9]. Notably, CrossFit was indicated as the primary reason HIIT workouts were ranked so high [6–9]. However, a consensus paper produced by the Consortium for Health and Military Performance (CHAMP) and ACSM associated a potential emergence of a high injury risk with programs such as CrossFit [10]. While positive influences on body composition and physical fitness were recognized, the consensus highlighted a “disproportionate musculoskeletal injury risk from these demanding programs, particularly for novice participants, resulting in lost duty time, medical treatment and extensive rehabilitation”. In addition, the consensus suggested the existence of a training paradigm requiring advanced level technique during maximal timed exercise repetitions without adequate rest intervals between sets, as well as an insufficient recovery time between high-volume loads and training sessions. This overload situation can lead to early fatigue, additional oxidative stress, less resistance to subsequent repetitive exercise strain, greater perception of effort, and unsafe movement execution [10]. Furthermore, this training context associated with inadequate training load progression increases the risk of overuse injury, overreaching, and overtraining. The consensus authors suggested, as a possible solution, individual monitoring of training load to minimize these risks [10]. Despite the proposed risks of CrossFit, others have suggested that high-intensity functional training programs, including CrossFit, have similar or lower potential for injury than many traditional physical training activities [11]. However, the authors also stated that controlling training volume must be done in order to reduce injury risk in military populations. For an effective training process and adaptation to occur, the monitoring [12], quantification [13], and regulation [14] of training load is necessary. However, managing training load poses a considerable challenge for sport scientists [15, 16]. Despite this challenge, managing training load is fundamental to achieving the objectives of reducing injury risk and optimizing sports performance [17–22].

Although there are a large number of CrossFit participants, empirical evidence demonstrating the improvements in physical fitness that arise from this form of training are far from substantive. Furthermore, an overview of CrossFit’s outcomes has not been verified. Therefore, the purpose of the present study was to analyze the findings of the scientific literature related to CrossFit through a systematic review and meta-analysis.

## Methods

### Literature Search

**Fig. 1:**
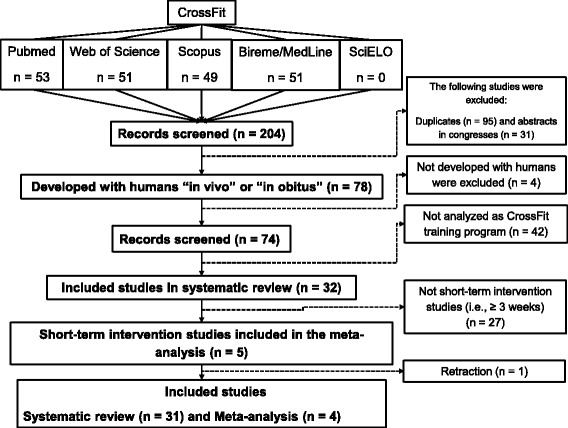
Study selection PRISMA flow diagram

One author conducted the literature search, collated the abstracts, and applied the initial inclusion criteria. The keyword “CrossFit” was used during the electronic search. The following electronic databases were searched on the 25th of November 2016: PubMed, Web of Science, Scopus, Bireme/MedLine, and SciELO (Fig. 1). The Preferred Reporting Items for Systematic Reviews and Meta-Analyses (PRISMA) reporting guidelines were adhered to in this manuscript. In the initial analysis, all CrossFit articles included in this manuscript were peer-reviewed and not limited to specific years or language. During the second phase of study selection, two authors reviewed and identified the titles and abstracts based on the inclusion criteria.

### Inclusion Criteria

To meet the inclusion criteria for the meta-analysis, studies investigating humans “in vivo” or “in obitus” and analyzed the effects of CrossFit as a training program were considered. The meta-analysis was only conducted on variables from short-term intervention studies (i.e., ≥ 3 weeks) with healthy male and/or female participants split into distinct gender groups (the procedures were consistent from those of another meta-analysis) [23]. Moreover, the variables analyzed were to be found in more than one study. If pertinent data were absent, authors were contacted and the necessary information requested via e-mail. If the original data were not provided by the authors, the mean and standard deviations were extracted from graphical representation using Ycasd [24] or estimated from the median, range, and sample size [25]. The remaining articles were included in the systematic review.

### Study Quality

The Consolidated Standards of Reporting Trials (CONSORT) statement was adapted and used for checking the quality of reporting by two authors independently. Thus, the articles’ quality was evaluated based on the 25 items identified in the CONSORT criteria, providing a maximal possible score of 37. The CONSORT items are distributed in sections and topics such as “Title and abstract”; “Introduction” (Background and objectives); “Methods” (Trial design, Participants, Interventions, Outcomes, Sample size, Blinding, Statistical methods); “Results” (Participant flow, Recruitment, Baseline data, Numbers analyzed, Outcomes and estimation, Ancillary analyses, Harms); “Discussion” (Limitations, Generalizability, Interpretation); and “Other information” (Registration, Protocol, Funding) [26]. Additionally, the Oxford Levels of Evidence [27] were used to evaluate the level of evidence for all articles found in the literature on CrossFit. Where the five levels (i.e., Level 1 = systematic reviews; Level 2 = randomized controlled trials with low/moderate risk of bias or observational studies with dramatic effect; Level 3 = cohort study, non-randomized controlled trials with low/moderate risk of bias or randomized controlled trial at high risk of bias; Level 4 = case series, case report, case-control studies, cohort study, historically controlled studies or non-randomized controlled trials at high risk of bias; and Level 5 = mechanism-based reasoning/expert opinion) are determined based on the following questions: (i) “How common is the problem?”; (ii) “Is this diagnostic or monitoring test accurate? (diagnosis)”; (iii) “What will happen if we do not add a therapy? (prognosis)”; (iv) “Does this intervention help? (treatment benefits)”; (v) “What are the COMMON harms? (treatment harms)”; (vi) “What are the RARE harms? (treatment harms)”; and (vii) “Is this (early detection) test worthwhile? (screening)”.

### Bias Analysis

For the systematic review, two authors independently assessed the quality of the included studies using the Cochrane risk of bias tool [28] with a priori formulated criteria adopted from the studies of Pas et al. [29] and Winters et al. [30]. Five domains of bias were appraised: selection bias (random allocation and allocation concealment), performance bias (blinding of personnel and participants), detection bias (blinding of outcome assessment), attrition bias (loss to follow-up), reporting bias (outcome reporting), and other biases. Each item was scored as low (+), high (−), or unclear (?) risk of bias. Studies were considered low risk of bias when all domains were scored as low risk of bias or if one item was scored as high risk or unable to determine. If two domains were scored as high or unable to determine risk of bias, the study received a moderate risk of bias. Finally, when more than two domains were scored as high risk of bias, the study was regarded to possess a high risk of bias. In case of disagreement between authors, consensus was sought during a consensus meeting. If no consensus was reached, a third author was asked to provide a final verdict. Publication bias was determined for the meta-analysis using an approach where differences in baseline assessments were checked for all intervention groups. Next, the interventions were divided into non-significant (*p* > 0.05) or significant (*p* < 0.05) results to determine the percentage of interventions with non-significant differences (these procedures were followed as per another meta-analysis) [23].

### Statistical Analysis

For the meta-analysis, the heterogeneity of the included studies was evaluated by examining forest plots, confidence intervals (CI), and *I*^2^. *I*^2^ values of 25, 50, and 75 indicated low, moderate, and high heterogeneity, respectively [31]. Random effects were analyzed using the DerSimonian and Laird [32] approach. The meta-analysis was conducted based on the number of variables from short-term intervention studies. Statistical significance was set at *p* ≤ 0.05, and the magnitude of differences for each dependent variable was calculated using effect size (ES) with 95% CI [32]. The ES classification was large > 0.80; moderate = 0.20–0.80; small < 0.20 [33]. Inferential statistics were used for the descriptive analysis of the data. All data were analyzed using CMA v3 trial (Biostat, New Jersey, USA) and Excel 2010 worksheet (Microsoft, Washington, USA).

## Results

The initial search found 204 articles (Fig. 1). When the inclusion criteria were applied, 32 articles were included in the systematic review. When the inclusion criteria were applied for the meta-analysis, five of these articles met the criteria and were included in the manuscript [4, 5, 34–63]. However, during this manuscript peer-reviewing process, one of 32 articles had a retraction published [64].

**Fig. 2:**
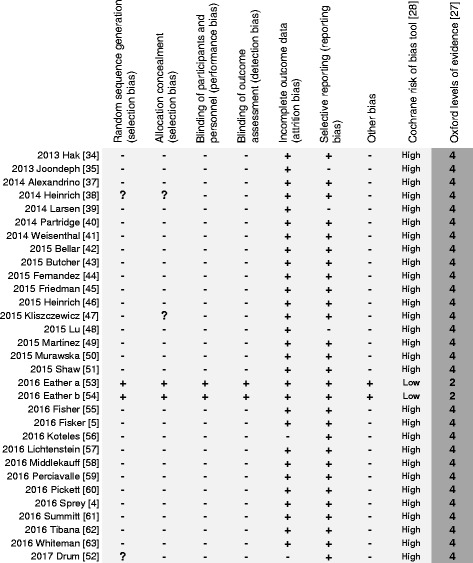
Risk of bias and level of evidence

Quality assessment of the 31 included articles ranged from 22 to 84% with a mean CONSORT rating of 37% [26]. Only 9% (i.e., absolute number = 3) of the included articles [38, 53, 54] had ratings exceeding 50% (Additional file 1: Table S1). Ethical approval was obtained in all articles. The evidence level ranged between levels 2 and 4 for included articles. However, just 6% (i.e., absolute number = 2) of articles were considered level 2 (i.e., randomized controlled trials with low risk of bias) (Fig. 2) [53, 54].

For the systematic review, only 6% of the assessed articles were at low risk of bias (Fig. 2) [53, 54]. These articles performed adequate randomization and allocation methods, blinding strategy, and clinical trial registry. In contrast, a majority of the non-controlled trials, cross-sectional studies based on an electronic questionnaire, and correlation studies or case report/case series did not explicitly describe if and how they controlled for detection bias. For the included articles in the meta-analysis, 78% of the intervention groups resulted in non-significant (*p* > 0.05) differences in baseline assessments (i.e., 83 interventions with non-significant differences ÷ 106 overall interventions = 78%).

The pooled sample size for this manuscript was 3597 with 81% of participants in the CrossFit group and the remaining 19% in the control group. Male participants (60%) were utilized more so than females (40%). CrossFit samples were composed of adolescents (male 4%, *n* = 112 and age = 15 ± 1 years; female 3%, *n* = 94 and age = 15 ± 1 years), adults (male 56%, *n* = 1638 and age = 30 ± 7 years; female 37%, *n* = 1065 and age = 30 ± 7 years), and elderly (male 0.2%, *n* = 5 and age > 60 years; female 0.1%, *n* = 2 and age > 60 years). The sample profile included 6% competitors (i.e., in the CrossFit Games), 63% trained individuals (i.e., in the CrossFit program more than 6 months), 22% physically active individuals, and 9% sedentary individuals. The average duration of each CrossFit intervention was 9 ± 3 weeks.

In summary, the following aspects of CrossFit were examined in the scientific literature: body composition (*n* = 4), psycho-physiological parameters (*n* = 12), musculoskeletal injury risk (*n* = 7), life and health aspects (*n* = 4), and psycho-social behavior (*n* = 11) (Table 1).

**Table 1:** Main findings of CrossFit’s scientific state of the art

Article (1^st^ author)	Aspects (type)	Sample (profile; *n*)	Intervention or method of analysis	Experimental design	Main findings
2013 Hak [34]	Injury risk	Trained people (*n* = 132)	By electronic questionnaire with people who had trained in CrossFit affiliates	Descriptive epidemiological study	74% of practitioners had suffered at least one injury while practicing CrossFit. The most common injury sites were the shoulder, lower back, and followed by arm/elbow with an injury rate of 3.1 per 1000 h.
2013 Joondeph [35]	Injury risk	Trained person (*n* = 1)	Traumatic retinal detachment occurring as result of CrossFit workout doing pull-ups with an elastic band tied around his waist and secured to the pull-up bar thus partially supporting his weight	Case report	Retina was successfully recovered and vision was normal after 4 months of follow-up.
2014 Alexandrino [37]	Life and health	Trained person (*n* = 1)	Sports-related stroke registries	Case series	A case of stroke type intracerebral hemorrhage during CrossFit training with follow-up of 4 months. Study conclusion confirmed that stroke during sport activity is rare and occurs mostly in heathy young males.
2014 Heinrich [38]	Body composition and psycho-social behavior	Sedentary (*n* = 20)	8 weeks of CrossFit training	Chronic effects	CrossFit practitioners were able to maintain exercise enjoyment and were more likely to intend to continue. No significant changes in body composition were found.
2014 Larsen [39]	Life and health	Trained person (*n* = 1)	Sports-related rhabdomyolysis registry	Case report	CrossFit practitioner had reported a rhabdomyolysis diagnostic after CrossFit training.
2014 Partridge [40]	Psycho-social behavior	Trained people (*n* = 144)	By electronic questionnaire with people who had trained in CrossFit affiliates	Descriptive epidemiological study	The inclusivity is highlighted in CrossFit. However, motivational climate and goals in CrossFit may vary based on demographic variables (i.e., gender and length of time in a program) and that these differences may impact how to most effectively motivate, encourage, and instruct group members.
2014 Weisenthal [41]	Injury risk	Trained people (*n* = 381)	By electronic questionnaire with people who had trained in CrossFit affiliates	Descriptive epidemiological study	19% of practitioners had suffered at least one injury while practicing CrossFit. the shoulder and lower back were the most commonly injured in gymnastic and power lifting movements, respectively.
2015 Bellar [42]	Physiological	Competitors (*n* = 21) and physically active (*n* = 11)	Session 01: 12 throws of a 9.07-kg medicine ball at a 3.05-m target, 12 swings of a 16.38-kg kettlebell, and 12 burpee pull-ups (AMRAP during 12 min)	Correlational study	AMRAP workout performance was associated with both aerobic fitness and anaerobic power.
			Session 02: sumo deadlift high pull, 0.5-m box jump, and 40-m farmer’s walk gripping two 20 kg bumper plates (21/15/9 = 21 repetitions in round one, 15 repetitions in round two, and 9 repetitions in round three)		
2015 Butcher [43]	Physiological	Trained people (n = 14)	Grace: 30 clean and jerks for time	Correlational study	CrossFit benchmark WOD performance cannot be predicted by VO_2max_, Wingate power/capacity, or either respiratory compensation or anaerobic thresholds.
			Fran: three rounds of thrusters and pull-ups for 21, 15, and 9 repetitions		
			Cindy: 20 min of rounds of 5 pull-ups,10 push-ups, and 15 bodyweight squats		
			CrossFit total: 1 repetition max back squat, overhead press, and deadlift.		
2015 Fernandez [44]	Physiological	Trained people (*n* = 10)	Fran: three rounds of thrusters and pull-ups for 21, 15, and 9 repetitions	Acute effects	Both WODs could be characterized as high intensity workouts, achieving near maximal physiological (e.g., 90–95% of HR_max_; blood lactate values > 10 mmol^− 1^) and perceptual responses (e.g., RPE values > 8).
			Cindy: 20 min of rounds of 5 pull-ups,10 push-ups, and 15 air-squats		
2015 Friedman [45]	Injury risk	Trained person (n = 1)	Magnetic resonance imaging examination demonstrated a high-grade tear of the right latissimus dorsi myotendinous junction. Initial injury occurred while performing a muscle up exercise.	Case report	This competitor was treated conservatively and was able to resume active CrossFit training within 3 months. At 6 months post-injury, he had only a mild residual functional deficit compared with his pre-injury level.
2015 Heinrich [46]	Body composition, physiological and psycho-social behavior	Sedentary (*n* = 6)	5 weeks of CrossFit training	Chronic effects	CrossFit training to cancer survivors had provoked significant improvements in emotional functioning, body composition (i.e., lean mass, fat mass and body fat percentage), balance, carrying a weighted object, lower body strength and power, aerobic capacity and endurance, and perceived difficulty for flexibility.
2015 Kliszczewicz [47]	Physiological	Physically active (*n* = 10)	Cindy: 20 min of rounds of 5 pull-ups,10 push-ups, and 15 air-squats	Acute effects	The Cindy bout elicited an acute blood oxidative stress response comparable to a traditional bout of high-intensity treadmill running.
2015 Lu [48]	Life and health	Trained people (*n* = 3)	Magnetic resonance imaging examination demonstrated the cervical internal carotid artery dissection	Case report	While direct causality cannot be proven, intense CrossFit workouts may have led to the internal carotid artery dissections in these practitioners.
2015 Martínez [49]	Psycho-social behavior	Physically active (*n* = 104)	8 CrossFit sessions during a didactic unit in the school	Chronic effects	CrossFit practice during physical education lessons have shown high levels of enjoyment, effort, and learning perception in the students. Furthermore, boys perceive higher enjoyment and intensity than girls.
2015 Murawska [50]	Body composition and physiological	Physically active (*n* = 12)	12 weeks of CrossFit training	Chronic effects	CrossFit training had beneficial influence on the practitioners’ body composition, anaerobic capacity and cardiovascular fitness as well as an increase in brain-derived neurotrophic factor (a protein that stimulates processes of neurogenesis).
2015 Shaw [51]	Physiological	Sedentary (*n* = 12)	CrossFit triplet: 3 burpees, 4 push-ups, and 5 squats	Acute effects	This WOD can be considered moderate to high intensity (heart rate ~ 108 bpm; blood lactate ~ 6 mmol/L) and is of sufficient intensity and safety (no significant changes in blood pressure, blood glucose, total cholesterol, and pulse and arterial pressure).
2016 Eather [53]	Psycho-social behavior	Physically active (*n* = 51)	8 weeks of CrossFit teens training	Chronic effects	CrossFit teens training did not improve mental health outcomes in the full students. However, the results from this study provides preliminary evidence for improving mental health in adolescents “at risk” of developing psychological disorders.
2016 Eather [54]	Body composition, physiological and psycho-social behavior	Physically active (*n* = 51)	8 weeks of CrossFit teens training	Chronic effects	CrossFit teens training had improved body composition (i.e., waist circumference, BMI) and results in performance tests (i.e., sit and reach, standing jump, and shuttle run). Retention was 82%, adherence was 94%, and satisfaction ranged from 4.2 to 4.6 out of 5 (1 = strongly disagree to 5 = strongly agree)
2016 Fisher [55]	Psycho-social Behavior	Trained people (*n* = 314)	By electronic questionnaire with people who had trained in CrossFit affiliates, group resistance exercise, alone and personal trainer	Descriptive epidemiological study	The study findings suggest that the motivations for engaging in CrossFit may be similar to those seen in sport participation and therefore may have an influence on facilitating long-term adherence in comparison with other resistance exercise modalities.
2016 Fisker [5]	Biomechanical	Trained people (*n* = 34)	5 rounds, 5 front squats; 10 box jumps; 15 double unders	Acute effects	Increased thickness of patellar and Achilles tendons, without changes in Plantar.
2016 Koteles [56]	Psycho-social behavior	Trained people (*n* = 186)	By electronic questionnaire with people who had trained in CrossFit affiliates	Descriptive epidemiological study	CrossFit training was not connected with higher levels of psychological functioning (well-being, affect, body awareness, and self-esteem) and satisfaction with body image.
2016 Lichtenstein [57]	Psycho-social behavior	Trained people (*n* = 598)	By electronic questionnaire with people who had trained in CrossFit affiliates	Descriptive epidemiological study	This study found a prevalence of exercise addiction of 5% in CrossFit. Exercise addiction is more prevalent in young practitioners (below 30 years) and in males. It is associated with high exercise volumes and negative exercise attitudes that might lead to negative consequences such as injuries and loss of social relations.
2016 Middlekauff [58]	Life and health	Physically active (*n* = 70)	CrossFit: 15 push-ups, 5 deadlifts at 80% of 3 repetition maximum, 5 push-presses at 80% of 3RM, 15 burpees, and 20 sit-ups	Acute and chronic effects	Acute: after an exercise bout typical for each group, vaginal support and vaginal resting pressure decreased slightly in both groups.
			Walking: 20-min walk at their self-selected exercise pace		Chronic: the strenuous exercise did not promote beneficial or deleterious effects for nulliparous women. Pelvic floor muscle strength did not change.
2016 Perciavalle [59]	Physiological	Competitors (*n* = 15)	WOD 15.5: Thrusters + rowing with 29/27/15/9 repetitions	Acute effects	High levels of blood lactate negatively impacted the performance of dual task attention and reaction time.
2016 Pickett [60]	Psycho-social behavior	Trained people (*n* = 276)	By questionnaire with people who had trained in CrossFit affiliates, group exercises, or individual exercise programs	Descriptive epidemiological study	The study found that the explicit community-building mantra encouraged by CrossFit was successful in creating greater levels of felt sense of community than other fitness outlets.
2016 Sprey [4]	Injury risk	Trained people (*n* = 622)	By electronic questionnaire with people who had trained in CrossFit affiliates	Descriptive epidemiological study	31% of practitioners had experienced some type of injury while practicing CrossFit.
2016 Summitt [61]	Injury risk	Trained people (*n* = 187)	By electronic questionnaire with people who had trained in CrossFit affiliates	Descriptive epidemiological study	24% of practitioners had suffered at least one shoulder injury in the last 6 months. Injury rate was 1.9 per 1000 h.
2016 Tibana [62]	Physiological	Trained people (*n* = 9)	WOD 01: 5× snatch (80% 1MR with 2–5 min of rest); 3 × 5 of touch and go snatches full (75% 5MR with 90 s of rest); 3 × 60 s of weighted plank hold (90 s of rest); after 5 min of rest: 10 s of as many round as possible (AMRAP) of 30 double unders and 15 power snatches (34 kg). WOD 02: 5× clean and jerk box (80% 1MR with 2–5 min of rest); 3 × 5 of touch and go cleans full (70% 5MR with 2–5 min of rest); 3 × 10 of strict hand standing push-ups; after 5 min of rest: 12 min of AMRAP of row 250 m and 25 target burpees	Acute effects in 2 consecutive days	Increases in blood glucose and lactate levels, along with pro and anti-inflammatory cytokines but without interfering in muscle performance for the next training session.
2016 Whiteman [63]	Psycho-social behavior	Trained people (*n* = 100)	By questionnaire with people who had trained in CrossFit affiliates and traditional gym	Descriptive epidemiological study	The study found that CrossFit may offer a greater sense of community level compared with a traditional gym. Specifically, CrossFit practitioners had higher levels of social capital and feelings of community belongingness than members of a similar traditional gym.
2017 Drum [52]	Physiological, life, and health	Trained people (*n* = 157)	By electronic questionnaire with people who had trained in CrossFit affiliates and ACSM-certified personal trainers clinics	Descriptive epidemiological study	CrossFit was perceived as strenuous or “very hard” activity by practitioners as well as they have been reporting a feeling of excessive fatigue, muscle pain and swelling, and limb movement difficulties within 48 h after a workout. A practitioner was diagnosed with rhabdomyolysis.

*ACSM* American College of Sports Medicine, *AMRAP* as many rounds as possible, *BMI* body mass index, *bpm* beats per minute, *HR*_*max*_ maximum heart rate, *mmol/L* millimole/liter, *MR* maximum repetitions, *RPE* ratings of perceived exertion, *VO*_*2max*_ maximal oxygen uptake, *WOD* workout of the day

Among the included short-term intervention studies, five CrossFit fitness domains were found in the literature, i.e., cardiovascular/respiratory endurance [50, 53], stamina [50, 53], strength [53], flexibility [53], and power [50, 53]. Five domains were yet to be verified, i.e., speed, coordination, agility, balance, and accuracy.

**Fig. 3:**
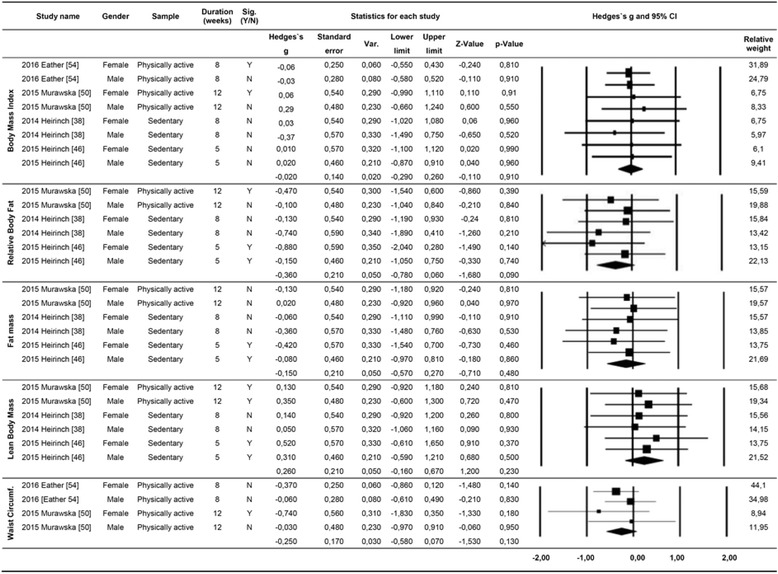
Meta-analysis of short-term intervention studies

Forty-three variables were found from short-term intervention studies in the meta-analysis. These variables represented cardiovascular/respiratory endurance and stamina (i.e., absolute and relative maximal oxygen consumption, VO_2max_), as well as body composition (i.e., body mass, body mass index, relative body fat, fat mass, lean body mass, and waist circumference). No significant results were found for any of the variables (Fig. 3).

## Discussion

Although CrossFit has a large number of participants, a high level of evidence demonstrating positive outcomes has yet to be established in the literature. Therefore, the present study aimed to verify the findings of scientific investigations related to CrossFit fitness domains as well as present outcome validity of CrossFit via systematic review and meta-analysis. Five of ten CrossFit fitness domains (cardiovascular/respiratory endurance, stamina, strength, flexibility, and power) were found in short-term intervention studies, with the remaining fitness domains (speed, coordination, agility, balance, and accuracy) lacking. Furthermore, CrossFit’s outcome evidence was provided for studies examining body composition, psycho-physiological parameters, musculoskeletal injury risk, life and health aspects, and psycho-social behavior. With respect to these studies, few achieved a high level of evidence at low risk of bias.

Meta-analyses were performed on body composition parameters including body mass index, relative body fat, fat mass, lean body mass, and waist circumference. All variables had non-significant results, reinforcing the need for more high-quality studies on CrossFit as well as long-term interventions.

### Psycho-physiological Parameters

A study comparing CrossFit training with a training approach based on ACSM recommendations reported CrossFit training as more strenuous and considered a “very hard” activity by participants [52]. CrossFit participants also reported greater fatigue, greater muscle pain and swelling, and limb movement difficulties during or within 48 h after the workout [52]. Furthermore, the authors reported the five most frequently used and hardest WODs were “Fran,” “Murph,” “Fight Gone Bad,” “Helen,” and “Filthy Fifty.” Except for “Fran,” the psycho-physiological responses to these WODs were not reported. “Fran” and another popular WOD known as “Cindy” presented greater magnitudes for heart rate (95–97% of HR_max_), %VO_2max_ (57–66%), blood lactate (14–15 mmol/L), and rate of perceived exertion (RPE) [44]. Perciavalle et al. [59] also observed lactate concentrations around 14 mmol/L following a WOD called “15.5”. “Cindy” (98% HR_max_ and RPE = 9) also presented an acute blood oxidative stress response similar to a traditional bout of high-intensity treadmill running (running at a minimum intensity of 90% maximum heart rate over 20 min) [47].

Researchers have reported a decrease in anti-inflammatory cytokines without decrements in muscle power following two consecutive days of CrossFit training sessions [62]. The WODs employed included a rest interval between sets and exercises (i.e., 2–5 min, for more details see Table 1). Thus, IL-6 displayed an increase immediately after training WOD 1 and WOD 2 while IL-10 displayed an increase immediately after WOD 1 only and decreased 24 and 48 h following WOD 2 when compared to baseline values [62]. These findings should be considered with caution as while there are designated rest intervals in some CrossFit workouts (e.g., Fight Gone Bad, 5 × 500 m row), the inclusion of rest intervals is not common practice in CrossFit prescriptions.

In an acute study, the WOD “CrossFit triplet” (i.e., three burpees, four push-ups, and five squats; for details see Table 1) was associated with significant changes in physiological responses [51]. Participants achieved approximately 12,000 mmHg for rate pressure product, 6 mmol/L for blood lactate, and 54% of HR_max_ [51]. According to the authors, “CrossFit triplet” was of moderate to high intensity and thus considered a viable interval training option that provides sufficient intensity in a safe manner [51].

In the correlation studies, whole-body strength, power, endurance, and experience seemed to be important measures associated with performance in CrossFit [42, 43]. Butcher et al. [43] reported whole-body strength as a predictor of performance in some WODs such as “Grace,” “Fran,” and “Cindy”. The authors also found VO_2max_, Wingate power, and anaerobic thresholds were unsuccessful in predicting WOD performance. Conversely, Bellar et al. [42] found VO_2max_ and anaerobic power to be significant predictors of performance after one CrossFit training session. The authors also divided 32 young healthy men into two groups and found CrossFit experience, or CrossFit training history, was also a predictor of performance during a WOD. Nonetheless, more research is required as the present literature is inconclusive regarding predictors of CrossFit performance.

Based on the systematic review, in general, WODs present highly varied psycho-physiological demands: heart rate between 54 and 98% of HR_max_, blood lactate levels between 6 and 15 mmol/L, %VO_2max_ between 57 and 66%, RPE between 8 and 9 (out of 10), and rate pressure product around 12,000 mmHg. Some WODs (e.g., “Fran,” “Cindy,” and “15.5”) can be identified as high-intensity level whereas others (e.g., “CrossFit triplet”) can be considered moderate.

### Musculoskeletal Injury Risk

In one of the first publications on musculoskeletal injury risk, a descriptive epidemiological investigation used an electronic questionnaire to examine 132 CrossFit participants [34]. Results revealed 74% of CrossFit participants suffered at least one injury. The most common injury sites were shoulder and lower back followed by arm/elbow, with an injury rate of 3.1 events every 1000 h of training [34]. A total of 186 lesions were reported with some participants injured more than once in a period of 18 months. Nine of these cases required surgical intervention. In another study that examined the epidemiological profile of CrossFit participants, an injury prevalence of 31% was recorded [4]. In addition, when the participants were separated according to CrossFit experience, those who practiced CrossFit for more than 6 months (35%) showed significantly (*p* = 0.004) higher injury rates than those who practiced for less than 6 months (23%). This study also reported a 45% injury prevalence rate among athletes with more than 2 years of practice [4].

Another descriptive epidemiological study employed an electronic questionnaire to verify injury risk of the shoulder in CrossFit participants (*n* = 187). The authors found that 24% of participants reported at least one shoulder injury in the last 6 months of practice, with an injury rate of 1.9 per 1000 h. The most common attributed causes of injury were inadequate form of movement (33%) and exacerbation of previous injury (33%). Furthermore, 64% of those who suffered an injury reported a reduction in training for 1 month or less due to injury [61].

Similar electronic questionnaire and experimental approaches have also been used to examine injury risk in CrossFit (*n* = 381). Musculoskeletal injuries accounted for 19% of all injuries, with men injured more frequently than women (*p* = 0.03). The shoulder was injured most often during gymnastic movements whereas the lower back was injured most often during power lifting movements [41].

In addition, two case reports offered insight on injury risk. The first case study examined a traumatic tear of the latissimus dorsi myotendinous junction inflicted during the “muscle up” exercise [45]. This injury usually occurs in the acute configuration of forced abduction and external rotation during resisted contraction. Performing this exercise requires sound technique and high levels of strength, particularly at the transition point of the maneuver. The participant in this case report returned to complete pre-injury level of activity within 6 months after the inciting event, with mild residual functional deficit. The second case report was a retinal detachment due to CrossFit training [35]. A 25-year-old male presented an inferior scotoma in the right eye after engaging in a CrossFit workout which required “pull ups” with an elastic band tied around the waist and secured to the pull up bar, thus partially supporting body weight. The retina was successfully reattached, and vision was successfully recovered after 4 months.

The acute effects of high-intensity CrossFit training on tendon properties were evaluated via ultrasonography (*n* = 34). Thickness of the patellar and Achilles tendons increased significantly after the session. These findings suggest the high-intensity loads associated with concentric and eccentric muscle actions during CrossFit exercise may result in an increase in patellar and Achilles tendon thickness. However, long-term interventions are needed to investigate the effect of recovery between high-intensity sessions as a deterministic factor in altering the structure of biomaterials within tendons and the subsequent effects of changes in tendon morphology on risk of injury [5].

In summary, the number of injuries that affect CrossFit participants varies between 19 and 74% with 1.9–3.1 per 1000 training hours. In this sense, the percentage of injury is relatively high while the incidence of injuries per 1000 h is low. These results may reflect a sampling bias or inadequate management of training volume. Although higher training volume and perception of intensity have been found in CrossFit participants [49, 52], further studies directly comparing the injury rates of CrossFit with other ACSM-recommended training modalities are warranted.

The second aspect highlighted by the CHAMP and ACSM consensus was monitoring individual-specific training load and its potential to minimize injury risk [10]. Although the cause of injury is multifactorial, injury can result from the summation of load that imposes a force that exceeds the capacity of the biological tissue involved [65]. To attenuate this deleterious outcome, an integrated approach that incorporates individual-specific monitoring [12], quantification [13], and regulation [14] may aid in decreasing injury risk. Monitoring is defined as the verification of responses to the training loads performed that were previously planned by the coach [12]. Quantification is defined as the sum of the training load that was effectively executed by the athlete [13]. Regulation is defined as the adjustments in the training loads lifted in relation to the athlete responses [14]. However, no studies investigating training load management were found in the systematic review, which presents a gap in current knowledge. Presently, controlling training load is based on the coach’s anecdotal and scientific background which can be highly varied around the world. Due to the potentially positive impact an evidence-based integrated approach to training load management could have on reducing injury, risk while achieving training objectives (i.e., enhancing sports performance) [17–22] warrants greater research in this area.

### Life and Health Aspects

Though sparse, case report and case series studies were found in the literature examining life and health aspects. Only two reported cases of rhabdomyolysis were found [39, 52]. However, this does not rule out the need to develop strategies of recovery between training sessions, respecting biological individuality of participants.

Other life and health aspects related to CrossFit training were found in the literature. Lu et al. [48] reported three cases of cervical carotid dissection that were associated with CrossFit workouts. Specifically, participant 1 suffered a distal cervical internal carotid artery dissection near the skull base and a small infarct in Wernicke’s area. The individual was placed on anticoagulation and on follow-up was near complete recovery. Participant 2 suffered a proximal cervical internal carotid artery dissection that led to arterial occlusion and recurrent middle cerebral artery territory infarcts, in addition to significant neurological sequelae. Participant 3 had a skull base internal carotid artery dissection that led to a partial Horner’s syndrome but no cerebral infarct. None of the three individuals died. While direct causality cannot be proven, the authors speculated the high-intensity CrossFit workouts likely led to the internal carotid artery dissections in these participants.

Similarly, Alexandrino et al. [37] examined 10 cases of stroke in participants aged between 27 and 65 years (80% being male). Among them, one man (32 years old) had an intracerebral hemorrhage stroke during a CrossFit session. The participant did not die, but he was left disabled ( no. 3 in the modified Rankin scale = moderate disability; requiring some help, but able to walk without assistance). The authors’ conclusion was that stroke during sport activity is rare, occurring mostly in healthy young males and that cervicocerebral arterial dissection is the primary mechanism of stroke, often without an explicit history of trauma.

Finally, researchers demonstrated neither beneficial nor deleterious effects on pelvic floor strength or support in nulliparous young women after CrossFit training [58]. The majority of these studies were evidence level 4 with high risk of bias and, as such, did not permit any recommendation.

To date, no studies have examined the effect of CrossFit training on resting blood pressure or heart rate. Further research examining the acute and chronic effects of CrossFit training on these health indicators is warranted.

### Psycho-social Behavior

A greater sense of community in CrossFit sessions was verified when compared to traditional training whether in a group or analyzed on an individual basis. This social interaction level was assessed via questionnaire in physically active participants [60, 63]. However, sense of community was not related to participant retention/adherence for any of the modalities analyzed [63].

The retention/adherence of participants was assessed in a randomized intervention study involving obese individuals (BMI > 30). The same number of dropouts was also revealed after 8 weeks of traditional training when compared to CrossFit with aerobic and resistance training. Nonetheless, the intention for continuing physically vigorous activity was greater for the CrossFit group [38]. Furthermore, a European Organization for Research and Treatment of Cancer core 30-item questionnaire revealed 5 weeks of CrossFit training was well received by cancer survivors with an adherence rate of 75%. This intervention was also considered feasible and effective in improving emotional function [46].

Motivation for the practice of physical activity was also assessed by questionnaire in four groups: CrossFit, resistance exercise, alone, and in individuals who train with a personal trainer. Enjoyment, challenge, and affiliation were identified in the CrossFit group more than all other training groups. Such source of motivation is compatible with that presented in sports practice. Individuals who trained with a personal trainer had higher health-related motives. However, this group was older than the other groups, which may represent a confounding factor in the response [54].

In schoolchildren (i.e., 12 to 16 years) participating in CrossFit exercise, an older age has been associated with higher ratings of perceived intensity and less enjoyment. In the between-sex comparison, boys perceived greater intensity and enjoyment [49]. Among adults, no sex difference was identified for the perceived motivational climate of CrossFit sessions, although the achievement goals varied between males and females [40]. With respect to achievement goals, the mastery-based motivational climate is initially predominant, but when a domain of the tasks is reached, the performance approach becomes predominant. These variations are also present between sexes, with females emphasizing mastery avoidance (i.e., to do as well as I can) and males emphasizing the performance approach (i.e., to do better than others) [40].

Although the goals within CrossFit practice vary with practice time, the same does not appear to be true for psychological functioning as well-being, affection, body awareness, and self-esteem were not influenced by the time or frequency of CrossFit practice [56]. Similar results were found in an 8-week intervention study in adolescent students (i.e., 15 years), where no improvement in mental health was observed. However, a subgroup of individuals at risk of psychological distress presented significant improvements in mental health [53]. In another study of the same research group, high levels of retention (i.e., 82%), adherence (i.e., 94%), and satisfaction (4.2–4.6 where 5 is the highest level) were found in the students after 8 weeks of CrossFit Teens training [54].

Lastly, CrossFit’s motivational characteristics, which aim to lead the individual to achieve the best performance possible, generated a 5% prevalence of exercise addiction in CrossFit participants which is similar to other exercise programs. This observation has also been shown to be even greater in men and young individuals (i.e., < 30 years). Exercise addiction was associated with a tendency to exercise despite injury, feelings of guilt when unable to exercise, passion turning into obsession, and taking medication to be able to exercise. These negative attitudes toward exercise can facilitate damage, such as injuries and losses in social relations, within participants [57].

In summary, there is preliminary evidence of a higher sense of community, satisfaction, and motivation among CrossFit participants. However, it is still necessary for new studies to verify the positive relationship between these factors and retention/adherence of participants.

### Brief Statement

Before finalizing, we wish to emphasize that this study did not seek to define CrossFit as “bad” or “good.” Rather, this investigation sought to present the possible benefits and risks associated with CrossFit according to current findings in the scientific literature. The low level of evidence at high risk of bias revealed by this study does not allow a stronger position on the advantages and disadvantages of CrossFit. The authors believe this disparity demonstrates the need to improve current methodological approaches in further studies, thus influencing current practice.

## Conclusions

Until now, current CrossFit scientific literature has been based on studies that investigated the effects of CrossFit on body composition, psycho-physiological parameters, musculoskeletal injury risk, life and health aspects, and psycho-social behavior. Meta-analysis did not find a significant effect of CrossFit training changes in body mass index, relative body fat, fat mass, lean body mass, and waist circumference. Unfortunately, the number of studies investigating CrossFit with high level of evidence at low risk of bias is sparse. As a result, these findings neither firmly establish the benefits or risks of CrossFit, nor provide definitive practical recommendations concerning CrossFit training. Despite this disparity, there is the existence of initial evidence of higher levels of sense of community, satisfaction, and motivation among CrossFit participants.

## Additional file

Additional file 1: **Table S1.** The Consolidated Standards of Reporting Trials (CONSORT). (DOCX 43 kb)
